# Granulomatosis With Polyangiitis Mimicking Temporal Arteritis

**DOI:** 10.1155/2024/9699571

**Published:** 2024-10-17

**Authors:** Ali Dehghan, Mahya Sadat Emami Meybodi, Shokoofeh Fooladmotlagh, Mohsen Zaremehrjardi, Hamidreza Soltani

**Affiliations:** ^1^Division of Rheumatology, Shahid Sadoughi University of Medical Sciences, Yazd, Iran; ^2^Department of Internal Medicine, School of Medicine, Shahid Sadoughi University of Medical Sciences, Yazd, Iran; ^3^Department of Rheumatology, School of Medicine, Shahid Sadoughi University of Medical Sciences, Yazd, Iran

**Keywords:** granulomatosis with polyangiitis, pachymeningitis, temporal arteritis

## Abstract

This case represents the first diagnosis of pachymeningitis due to granulomatosis with polyangiitis (GPA) in an elderly Iranian man who initially presented with persistent daily headaches. PCR tests of cerebrospinal fluid for tuberculosis, brucellosis, and fungal infections all yielded negative results. Given the pachymeningitis pattern observed on brain MRI and the absence of infectious and lymphoma diseases, along with positive anti-PR3 and proteinuria (793 mg in a 24-h urine sample), a diagnosis of GPA was established. The patient was treated with five doses of pulse methylprednisolone and one dose of pulse cyclophosphamide (1 g). Additionally, prednisolone 60 mg daily, monthly pulse cyclophosphamide, a daily calcium-D tablet, and alendronate 70 mg weekly were prescribed. Subsequently, the patient's headaches, hearing loss, and vision loss were completely resolved. GPA should be considered in older individuals with persistent daily headaches, especially when pachymeningitis is evident. The use of contrast-enhanced brain MRI is an essential diagnostic tool in such cases.

## 1. Introduction

Granulomatosis with polyangiitis (GPA), formerly known as Wegener's granulomatosis (WG), is a rare systemic disease with an annual incidence of 8.4 per million in the general population [1]. It is characterized by necrotizing granulomatous inflammation and vasculitis, with its classic form primarily affecting the upper and lower respiratory tracts and kidneys [2]. Central nervous system involvement is rare in patients with GPA and is frequently associated with dysfunction of one or more cranial nerves, with the second, third, sixth, and seventh nerves being the most commonly affected [3]. Pachymeningitis (PM) is a disorder characterized by localized or diffuse thickening of the intracranial or spinal dura mater. It is a rare finding in GPA [1], occurring in 0.6%–8% of these patients, typically accompanying localized disease. Some features are attributable to increased intracranial pressure, leading to severe headaches that may be refractory to analgesics or nonsteroidal anti-inflammatory agents, although improvement can occur with glucocorticoids. Other manifestations include cranial nerve palsies (resulting from nerve compression), meningism, seizures, encephalopathy, and paraplegia. Gadolinium (GAD)-enhanced brain magnetic resonance imaging (MRI) is the preferred diagnostic technique for PM [4]. To the best of our knowledge, there has been no previous report of GPA presenting with PM in our country. Therefore, this study reports a case of GPA presenting with PM in an elderly Iranian man.

## 2. Case Report Presentation

The patient, a 70-year-old man with no history of previous illnesses, was diagnosed with temporal arteritis eight months before the current visit due to sudden vision loss in the right eye, headache, fever, and anterior ischemic optic neuropathy (AION). He had been prescribed prednisolone 20 mg every other day and azathioprine 100 mg daily. The patient experienced severe headaches, predominantly on the right side, which did not intensify with exposure to light and sound stimuli. Analgesics provided only transient relief, lasting less than 1 hour. The patient was prescribed pethidine 25 mg every 8 h and oxycodone 2.5 mg every 12 h. Due to inadequate response, the oxycodone dosage was increased to 5 mg every 12 h (from 29 November 2023 until 3 December 2023).

Despite partial improvement with drug treatment, the vision in the right eye deteriorated again, and hearing loss manifested as an additional symptom.

During the second ophthalmological examination, a positive bilateral relative afferent pupillary defect (RAPD) [for right eye] was observed, attributed to advanced bilateral glaucoma, as this condition can also cause a positive RAPD. Treatment was initiated with Zilomol and brimonidine eye drops, and the patient was referred for outpatient glaucoma surgery.

Laboratory tests showed increased inflammatory markers [erythrocyte sedimentation rate (ESR): 59 mm/h, C-reactive protein (CRP): +2, white blood cell (WBC) count: 15.9 × 10^3^/*μ*L, hemoglobin (Hb): 11.1 g/dL, platelets (PLT): 511 × 10^3^/*μ*L].

In the ear, nose, and throat (ENT) examination, the auricles of both ears appeared normal, but wax was present in the ear canals, and the tympanic membrane was not visible. Despite attempts at washing, the patient's hearing did not improve. In addition, the headache improved.

### 2.1. BRAIN MRI With and Without GAD

**Figure 1:**
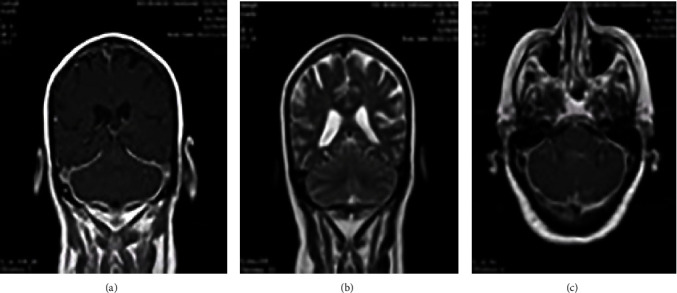
Diffuse smooth thickening and enhancement of the tentorium and adjacent basal dura were observed. Additionally, there was diffuse increased leptomeningeal enhancement in both supra and infra-tentorial parts of the brain, brain MRI, T1 sequence, and coronal view with GAD (a), T2 sequence and coronal view with GAD (b), T1 sequence and axial view with GAD (c).

Figure 1 shows diffuse smooth thickening and enhancement of the tentorium and adjacent basal dura were observed. Additionally, there was diffuse increased leptomeningeal enhancement in both supra and infra-tentorial parts of the brain, Brain MRI, T1 sequence and coronal view with GAD (a), T2 sequence and coronal view with GAD (b), T1 sequence and axial view with GAD (c).

Multiple bright foci were identified in T2 and FLAIR images in the white matter and periventricular areas, distributed across both cerebral hemispheres. This finding may be indicative of small vessel disease or ischemic lesions.

Upon receiving the MRI report suggesting PM potentially linked to chronic meningitis or lymphoma, the patient sought consultations with infectious disease and neurology specialists. Subsequently, a lumbar puncture (LP) and cerebrospinal fluid (CSF) analysis were recommended.

After consulting with a hematologist, the patient underwent a CT scan of the thorax, abdomen, and pelvis with intravenous contrast. Additionally, serum and urine protein electrophoresis and screening for urinary Bence Jones protein were performed.

**Figure 2:**
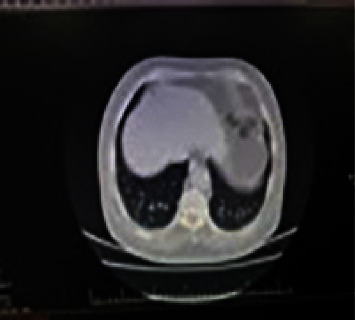
Small subpleural nodule in the right lower lobe (RLL), thoracic CT scan with intravenous contrast.

The CSF analysis revealed WBC = 5 × 10^3^/*μ*L, RBC = 0, glucose (GLU) = 77 mg/dL, lactate dehydrogenase (LDH) = 29 units/L, and protein (PRO) = 108 mg/dL. PCR tests for tuberculosis, brucellosis, and fungal infections all yielded negative results. Abdominal and pelvic ultrasound results were normal, and a small subpleural nodule was identified in the right lower lobe (RLL) during the thoracic CT scan with intravenous contrast (Figure 2).

**Figure 3:**
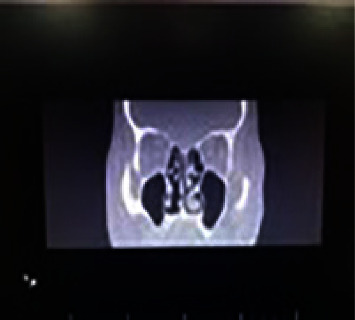
Normal paranasal sinuses CT scan.

To investigate paranasal sinus involvement, a CT scan was performed, which was normal (Figure 3). WRIGHT, 2-mercaptoethanol (2ME) tests, and Bence Jones urinary tests were all negative.

Echocardiography indicated an ejection fraction (EF) of 55%. A 24-h urine collection was conducted, resulting in a volume of 1300 cc, with a protein content of 793 mg and creatinine at 0.8 g/24 h. The reported values for C-antineutrophil cytoplasmic antibody (C-ANCA) and P-ANCA were 24.62 (normal range up to 18) and 0.23, respectively.

Considering the occurrence of hearing loss despite ongoing treatment and the relative improvement in blurred vision, which was not consistent with the AION pattern, the diagnosis of temporal arteritis became less certain. A blurred disc margin was observed in the right eye during the fundoscopic examination.

Given the PM pattern observed on the brain MRI, the negative results for infectious and lymphoma procedures, along with positive anti-PR3 and proteinuria (793 mg in a 24-h urine sample), a diagnosis of WG was made. The patient was treated with five doses of pulse methylprednisolone (1 g) and one dose of pulse cyclophosphamide (1 g). Additionally, prednisolone 60 mg daily, a daily calcium-D tablet, monthly pulse cyclophosphamide, and alendronate 70 mg weekly were prescribed, and the patient was discharged.

## 3. Discussion

Hypertrophic pachymeningitis (HP) is an inflammatory disorder characterized by thickening of the dura mater, both intracranially and within the spinal cord, leading to various neurological symptoms such as headaches, cranial neuropathies, seizures, and sensorimotor disturbances. One significant cause of immune-mediated HP is ANCA-associated vasculitis (AAV) [5]. Both IgG4-related disease (IgG4-RD) and idiopathic HP share similar demographic patterns, histopathological features, and natural histories, suggesting that IgG4-RD might be a common underlying cause of idiopathic HP [6]. In addition, PM associated with sarcoidosis typically presents with headaches, visual disturbances, and seizures. It often affects the dura mater in areas such as the falx cerebri, anterior and middle cranial fossae, and the tentorium cerebelli, and often necessitates treatment with steroid-sparing immunosuppressants [7].

Meningeal hypertrophic thickening has also been linked to diseases such as rheumatoid arthritis, syphilis, GPA (formerly WG), tuberculosis, cancer [8], and systemic lupus erythematosus [9].

Specifically, tuberculous PM though rare, should be considered in patients with chronic headaches, focal neurological symptoms, and MRI findings indicative of dural thickening [8].

AAV is implicated in the development of immune-mediated HP, with most cases seen in patients diagnosed with GPA [5].

In the current study, we diagnosed a case of GPA. The primary reported complaint was a right-sided headache. Another study highlighted that persistent and severe headaches often serve as the predominant and typical initial symptoms of meningeal involvement in GPA. Due to the frequent occurrence of headaches in GPA patients, often associated with chronic sinusitis or orbital disease, meningeal involvement might go unnoticed for an extended period. Neurological manifestations involving cranial nerves, particularly the second, third, sixth, and seventh nerves, have been frequently reported in patients with meningeal disease [2].

Higuera-Ortiz et al. also emphasized that headaches are a key feature of PM in GPA, particularly prevalent in cases with localized and predominant ENT disease. This characteristic may lead to the headache being misattributed to ENT involvement. However, especially when chronic, it could also indicate PM necessitating thorough investigation [4]. Onyeuku, Balakrishnan, and Cartwright assessed a 75-year-old male who initially presented with persistent daily headaches. After physical and lab examinations, they concluded that GPA should be considered in older patients presenting with persistent daily headaches and PM [10]. According to another study, the clinical manifestation of GPA with PM in a 46-year-old man included facial pain and headaches [11].

Early treatment of PM in GPA is associated with improved neurological outcomes. A positive response to immunosuppressive therapy characterized by the resolution of headaches, improvement or stabilization of cranial neuropathies and other neurological symptoms, reduction in ESR and CRP levels, and sometimes reversal of MRI abnormalities was observed in the majority of GPA-related PM cases [2]. In our study, the patient received 5 doses of pulse methylprednisolone (1 g each) and 1 dose of pulse cyclophosphamide (1 g). Additionally, prednisolone 60 mg, monthly pulse cyclophosphamide, daily calcium-D tablets, and weekly alendronate 70 mg were prescribed. The patient's headache and hearing loss completely resolved, and their vision also improved before discharge.

Sakellariou and Kefala reported a case of PM in GPA treated with daily prednisolone at 32 mg, gradually tapered, and seven monthly pulses of cyclophosphamide, along with 1 g of hydrocortisone. The patient exhibited prompt resolution of all symptoms, with mild residual sensorineural hearing loss in the left ear, improvement in abnormal laboratory tests, and moderate resolution of PM on follow-up brain MRI. Currently, the patient is receiving methotrexate at 20 mg weekly and prednisolone at 8 mg daily. Attempts to reduce prednisolone below 10 mg/day have resulted in headache recurrence, prompting consideration of rituximab treatment [2].

Li et al. presented a case involving a 66-year-old man with PM and aortitis as the initial presentation of GPA. The patient received intravenous methylprednisolone, followed by an oral prednisone taper. Rituximab was initiated, leading to near-resolution of neurological symptoms within 2 months. These findings suggest that PM can rarely be the initial manifestation of GPA, leading to cranial nerve impingement and venous outflow obstruction [12].

In a case reported by Just et al., a patient with GPA presenting with PM showed no clinical response to oral prednisolone and cyclophosphamide. However, complete clinical and imaging remission was achieved by adding rituximab to the treatment regimen [13]. Conversely, Kohlberg, Truong, and Chang reported symptomatic improvement in an adolescent with GPA presenting with PM after long-term treatment with prednisone and cyclophosphamide [1].

Based on these findings, the sensitivity of CT in detecting meningeal disease is generally low. In most cases, CSF examination reveals nonspecific abnormalities, such as mild pleocytosis primarily consisting of lymphocytes [2]. However, the widespread use of MRI has significantly enhanced the early identification and monitoring of patients with PM. A typical MRI finding includes thickening and contrast enhancement of the dura mater, with less frequent involvement of the leptomeninges. Two distinct MRI patterns were identified: (a) diffusely abnormal meninges unrelated to sinus or orbital disease and (b) focal enhancing thickening adjacent to sinus or orbital disease [2]. Onyeuku, Balakrishnan, and Cartwright also emphasized the importance of contrast-enhanced brain MRI as a crucial diagnostic tool, aligning with our findings [10].

## 4. Conclusion

GPA should be considered in older individuals with persistent daily headaches, especially when PM is evident. The use of contrast-enhanced brain MRI proves to be an essential diagnostic tool in such cases.

## Data Availability

Data sharing not applicable to this article as no datasets were generated or analyzed during the current study.

## References

[1] Kohlberg G. D., Truong M. T., Chang K. W. (2011). Wegener’s Granulomatosis in an Adolescent Presenting With Pachymeningitis, Mastoid Effusion and Horner’s Syndrome. *International Journal of Pediatric Otorhinolaryngol Extra*.

[2] Sakellariou G. T., Kefala N. (2013). Pachymeningitis in Granulomatosis With Polyangiitis: A Case Report and a Review of the Literature. *Case Reports in Rheumatology*.

[3] Jennette J. C., Falk R. J., Bacon P. A. (2013). 2012 Revised International Chapel Hill Consensus Conference Nomenclature of Vasculitides. *Arthritis & Rheumatism*.

[4] Higuera-Ortiz V., Reynoso A., Ruiz N., Delgado-Hernández R. D., Gómez-Garza G., Flores-Suárez L. F. (2017). Pachymeningitis in Granulomatosis With Polyangiitis: Case Series With Earlier Onset in Younger Patients and Literature Review. *Clinical Rheumatology*.

[5] Shimojima Y., Sekijima Y. (2023). Hypertrophic Pachymeningitis in ANCA-Associated Vasculitis: Clinical and Immunopathological Features and Insights. *Autoimmunity Reviews*.

[6] Wallace Z. S., Carruthers M. N., Khosroshahi A. (2013). IgG4-Related Disease and Hypertrophic Pachymeningitis. *Medicine*.

[7] Chakales P. A., Herman M. C., Chien L. C., Hutto S. K. (2022). Pachymeningitis in Biopsy-Proven Sarcoidosis: Clinical Course, Radiographic Findings, Response to Treatment, and Long-Term Outcomes. *Neurology(R) Neuroimmunology & Neuroinflammation*.

[8] Cordeiro N. L., Gupta S. S., Kanwar A., Bendor-Grynbaum C., Sharma J. B. (2021). Tuberculous Hypertrophic Pachymeningitis. *Cureus*.

[9] Patel J. (2019). Hypertrophic Pachymeningitis an Unusual Manifestation of Systemic Lupus Erythematosus: A Case Report. *Journal of the Neurological Sciences*.

[10] Onyeuku N. E., Balakrishnan N., Cartwright M. S. (2014). Granulomatosis With Polyangiitis Presenting With Pachymeningitis. *Journal of the Neurological Sciences*.

[11] Dörr J., Elitok S., Dieste F. J. (2008). Treatment-Resistant Chronic Headaches and Focal Pachymeningitis in a 46-Year-Old Man: A Rare Presentation of Wegener’s Granulomatosis. *The Lancet Neurology*.

[12] Li X., Stitt D., Lanzino G., Giannini C., Dubey D., Carabenciov I. D. (2023). Teaching NeuroImage: Pachymeningitis and Aortitis as the Initial Presentation of Granulomatosis With Polyangiitis. *Neurology*.

[13] Just S. A., Knudsen J. B., Nielsen M. K., Junker P. (2011). Wegener’s Granulomatosis Presenting With Pachymeningitis: Clinical and Imaging Remission by Rituximab. *ISRN Rheumatology*.

